# Implementing the Xpert® MTB/RIF Diagnostic Test for Tuberculosis and Rifampicin Resistance: Outcomes and Lessons Learned in 18 Countries

**DOI:** 10.1371/journal.pone.0144656

**Published:** 2015-12-15

**Authors:** Elisa Ardizzoni, Emmanuel Fajardo, Peter Saranchuk, Martina Casenghi, Anne-Laure Page, Francis Varaine, Cara S. Kosack, Pamela Hepple

**Affiliations:** 1 Médecins Sans Frontières, Institute of Tropical Medicine, Antwerp, Belgium; 2 Médecins Sans Frontières, Southern Africa Medical Unit, Cape Town, South Africa; 3 Médecins Sans Frontières, Geneva, Switzerland; 4 Epicentre, Médecins Sans Frontières, Paris, France; 5 Médecins Sans Frontières, Paris, France; 6 Médecins Sans Frontières, Amsterdam, Netherlands; 7 Médecins Sans Frontières, Manson Unit, London, United Kingdom; California Department of Public Health, UNITED STATES

## Abstract

**Background:**

The Xpert^®^ MTB/RIF (Xpert) is an automated molecular test for simultaneous detection of tuberculosis (TB) and rifampicin resistance, recommended by the World Health Organization as the preferred diagnostic method for individuals presumed to have multi-drug resistant TB (MDR-TB) or HIV-associated TB. We describe the performance of Xpert and key lessons learned during two years of implementation under routine conditions in 33 projects located in 18 countries supported by Médecins Sans Frontières across varied geographic, epidemiological and clinical settings.

**Methods:**

Xpert was used following three strategies: the first being as the initial test, with microscopy in parallel, for all presumptive TB cases; the second being only for patients at risk of MDR-TB, or with HIV- associated TB, or presumptive paediatric TB; and the third being as the initial test for these high-risk patients plus as an add-on test to microscopy in others. Routine laboratory data were collected, using laboratory registers. Qualitative data such as logistic aspects, human resources, and tool acceptance were collected using a questionnaire.

**Findings:**

In total, 52,863 samples underwent Xpert testing from April 2011 to December 2012. The average MTB detection rate was 18.5%, 22.3%, and 11.6% for the three different strategies respectively. Analysis of the results on samples tested in parallel showed that using Xpert as add-on test to microscopy would have increased laboratory TB confirmation by 49.7%, versus 42.3% for Xpert replacing microscopy. The main limitation of the test was the high rate of inconclusive results, which correlated with factors such as defective modules, cartridge version (G3 vs. G4) and staff experience. Operational and logistical hurdles included infrastructure renovation, basic computer training, regular instrument troubleshooting and maintenance, all of which required substantial and continuous support.

**Conclusion:**

The implementation of Xpert was feasible and significantly increased TB detection compared to microscopy, despite the high rate of inconclusive results. Xpert implementation was accompanied by considerable operational and logistical challenges. To further decentralize diagnosis, simpler, low-cost TB technologies well-suited to low-resource settings are still urgently needed.

## Introduction

Tuberculosis (TB) remains a major public health problem, as evidenced by the estimated 9 million incident cases, 300 000 multi-drug resistant (MDR) cases and 1.5 million deaths worldwide in 2013. [1] However, only 58% of the new incident cases were bacteriologically confirmed by smear, culture, or Xpert^®^ MTB/RIF (Xpert) (Cepheid, Sunnyvale, CA), while the remaining 42% were diagnosed clinically, including by X-ray. [2]

The Xpert test is an automated molecular system that allows for rapid, simultaneous detection of both *Mycobacterium tuberculosis* (MTB) and resistance to rifampicin, a key first-line anti-TB drug, directly from sputum [3] and extrapulmonary samples.[4] In 2010, the World Health Organization (WHO) endorsed the Xpert test and recommended its use as the initial diagnostic test for people with HIV-associated TB or presumptive multidrug resistant TB (MDR-TB).[5] Three years later the recommendation was extended (conditional on availability of resources) to cover initial diagnostic testing for all adults presumed of having TB.[6] The 2011 guidelines released by WHO described implementation of Xpert as simple, requiring only minimal staff training, and feasible in diverse settings.[7] These guidelines helped trigger rapid worldwide adoption of Xpert: as of June 2014, a total of 3,269 Xpert instruments had been procured for the public sector in 108 of 145 countries eligible for concessional pricing.[2]

The diagnostic accuracy of Xpert in different settings and patient populations has been confirmed by several extensive validation studies, including a multicentre study carried out in six countries [8] and two systematic reviews and meta-analyses.[9,10] However, nearly all these studies were conducted in controlled research environments that provide optimal conditions, and therefore may not reflect difficulties frequently encountered in routine programmatic contexts. Only one report, carried out in countries using different case detection strategies, reported on the performance of Xpert in a vast programmatic pilot project and provided comprehensive information on Xpert implementation under routine conditions. [11] However, the majority of the sites included used Xpert as an add-on test to microscopy, limiting the analysis of case detection provided by this technique as a first-line diagnostic test. [11]

In addition, a description of the difficulties experienced during the initial phases of technology implementation compared with a later stage of routine testing is still lacking. Yet knowledge of the common difficulties typically encountered during the initial implementation period in low-resource settings, and lessons learned in resolving them, are highly valuable to countries as they begin or continue scaling-up in new settings.

We present results from routine testing of pulmonary samples with Xpert following different diagnostic strategies adopted in TB programs supported by Médecins Sans Frontières (MSF). We also describe the key lessons learned during almost two years of implementation and routine use of Xpert under programmatic field conditions across varying ranges of geographic, epidemiological and clinical settings.

## Methods

### Study settings

From April 2011 to December 2012 a total of 38 Xpert four-module instruments were installed in 33 project sites in 18 countries supported by MSF, representing a diverse range of TB, MDR-TB and HIV prevalence. Sites were considered to have a high MDR-TB burden if the MDR prevalence previously reported in the patient cohort of the site exceeded 10% in newly diagnosed cases [12], while high HIV burden sites refers to settings with HIV prevalence ≥1%.[13]

Xpert devices were mainly placed in district and sub-district laboratories (21/33) apart from five regional, six peripheral and one penitentiary system facility. The site distribution of the Xpert instruments according to epidemiological setting and facility level per country is described in Table 1.

**Table 1:** Site distribution of GeneXpert instruments by epidemiological setting and facility level (n = 33).

	Site distribution by epidemiological setting				Distribution of sites by facility level			
	High MDR-TB /high HIV	High MDR-TB /low HIV	Low MDR-TB/ high HIV	Low MDR-TB/ low HIV	Regional	District or sub-district	Peripheral	Prison
Cambodia				1		1		
Central African Republic			1				1	
Colombia		1			1			
Democratic Republic of Congo			1			1		
Georgia		1			1			
India		1				1		
Kenya			3			2	1	
Kyrgyzstan		2				1		1
Lesotho	1					1		
Malawi			2			2		
Mozambique			2			1	1	
Myanmar		1			1			
Russia		1			1			
Somalia				1	1			
South Africa	2					2		
Swaziland	5					5		
Uzbekistan		2				1	1	
Zimbabwe			5			3	2	
TOTAL	8	9	14	2	5	21	6	1

### Laboratory procedures

Pulmonary samples collected from adults and children were tested with Xpert following the manufacturer’s instructions.[14] In children (defined as below 15 years of age) unable to produce sputum, alternative respiratory samples such as gastric aspirates and induced sputum were obtained where facilities and expertise to carry out these procedures existed. Xpert results were interpreted according to the manufacturer’s instructions. A test was reported as inconclusive if the Xpert instrument indicated a final automated result as invalid, error or no result which were not reported as desegregated data except for error 5011. In case of inconclusive results, Xpert was performed on the leftover sample or on a newly collected sample; however, these results were not provided for this data collection, which includes only results from the first testing. Initially, instruments at most sites used cartridge version G3. Version G4 was developed to reduce errors rate, mainly error 5011 rate, indicated by Cepheid as being due to signal loss detected in the amplification curve, and to improve test robustness by decreasing possible false rifampicin resistant results. This version was gradually introduced as it became available. [15]

Where culture techniques were available on site, samples were decontaminated and then tested with Xpert and inoculated in parallel either on Löwenstein–Jensen (LJ) culture medium, mycobacterial growth indicator tube (MGIT) (Becton Dickinson, Diagnostic Instrument Systems, Sparks, MD), or Thin Layer Agar (TLA) medium.

### TB testing strategies

The study sites employed three testing strategies. Xpert as the initial diagnostic test for all presumptive TB cases was adopted in 23 sites, 22 of them performing Xpert in parallel with microscopy. Nine sites subsequently dropped the use of microscopy after a minimum of 300 tests, which was then employed only for Xpert positive cases to obtain a smear baseline result for follow-up and infection control purposes. The second strategy was used at 7 sites, where Xpert use was limited to patients at high risk of MDR-TB (previously treated, non-converting patients, treatment failures, symptomatic contacts of confirmed MDR cases) [5], HIV-associated TB, and presumed paediatric TB cases. The third strategy was used at the remaining 3 sites, where Xpert was employed as the first test for high-risk groups plus as an add-on test to microscopy in others.

Three sites performed Xpert in parallel to culture, each site following one of the three different strategies.

### Data collection

#### Quantitative data and setting information

Data were collected between April 2011 and December 2012 by quarter and recorded in an electronic laboratory register developed for routine Xpert data collection. At each site, the period of use was divided into two phases: the “implementation phase”, covering the first four months of Xpert use and the “routine activity phase” covering the subsequent months of routine testing that were covered by the monitoring period. Projects also provided information on the month in which the use of new G4 cartridge version began. G4 was considered introduced from the middle of the month onwards. Additionally, each project reported any module replacement throughout the monitoring period.

All projects provided information about the facility level of the laboratory where Xpert was implemented, including a description of infrastructure, environmental conditions, workload in the laboratory and sample collection strategy.

#### Qualitative data

Xpert was introduced in the sites following a period of preparation, which included training for laboratory technicians and implementation of logistical requirements, according to manufacturer and WHO recommendations[7]. To identify the key lessons learned from implementing the GeneXpert system and Xpert testing from each study site, a questionnaire was distributed to all sites in December 2012, at which point the period of onsite Xpert testing ranged from four to 21 months. The questionnaire was completed by site laboratory coordinators. Questions focused on installation, daily use, maintenance of the equipment and calibration. Additional questions covered infrastructure requirements, requirement for training of laboratory staff, human resource issues related to the implementation of the GeneXpert system and overall impressions of GeneXpert instruments. The questionnaire requested answers in the form of yes/no, numbers, description of incidents, and included open responses.

### Statistical analysis

To determine the statistical significance in group comparisons, p-values were calculated using the chi-square or Fisher’s exact test for independent samples and McNemar’s test for matched-pair samples. Statistical tests were two-sided at alpha = 0.05, and p-values <0.05 were considered statistically significant. All analyses were performed using Stata version 11 (StataCorp, College Station, TX, USA).

Comparison between Xpert and smear microscopy results is presented as relative gain, expressed for Xpert as an add-on test (calculated as the number of Xpert-positive and smear negative specimens divided by the number of smear positive specimens), and Xpert as a replacement test for microscopy (calculated as the number of Xpert positive specimens minus the number of smear positive specimens divided by number of smear positive specimens).

### Ethics

The International Ethics Review Board of MSF reviewed the study protocol and determined that it did not require formal ethics board approval as it was based on retrospective analysis of routinely collected programmatic data.[16] None of the merged data can be linked to individuals and no patient identifiers were used, so confidentiality was preserved. The study did not require collection of additional patient samples or performance of any test additional to routine patient care procedures so the study did not constitute a risk for the patients. Due to the retrospective data analysis of merged results, the patients were not asked to provide informed consent.

## Results

### Detection of MTB complex in adult and paediatric samples

**Fig 1:**
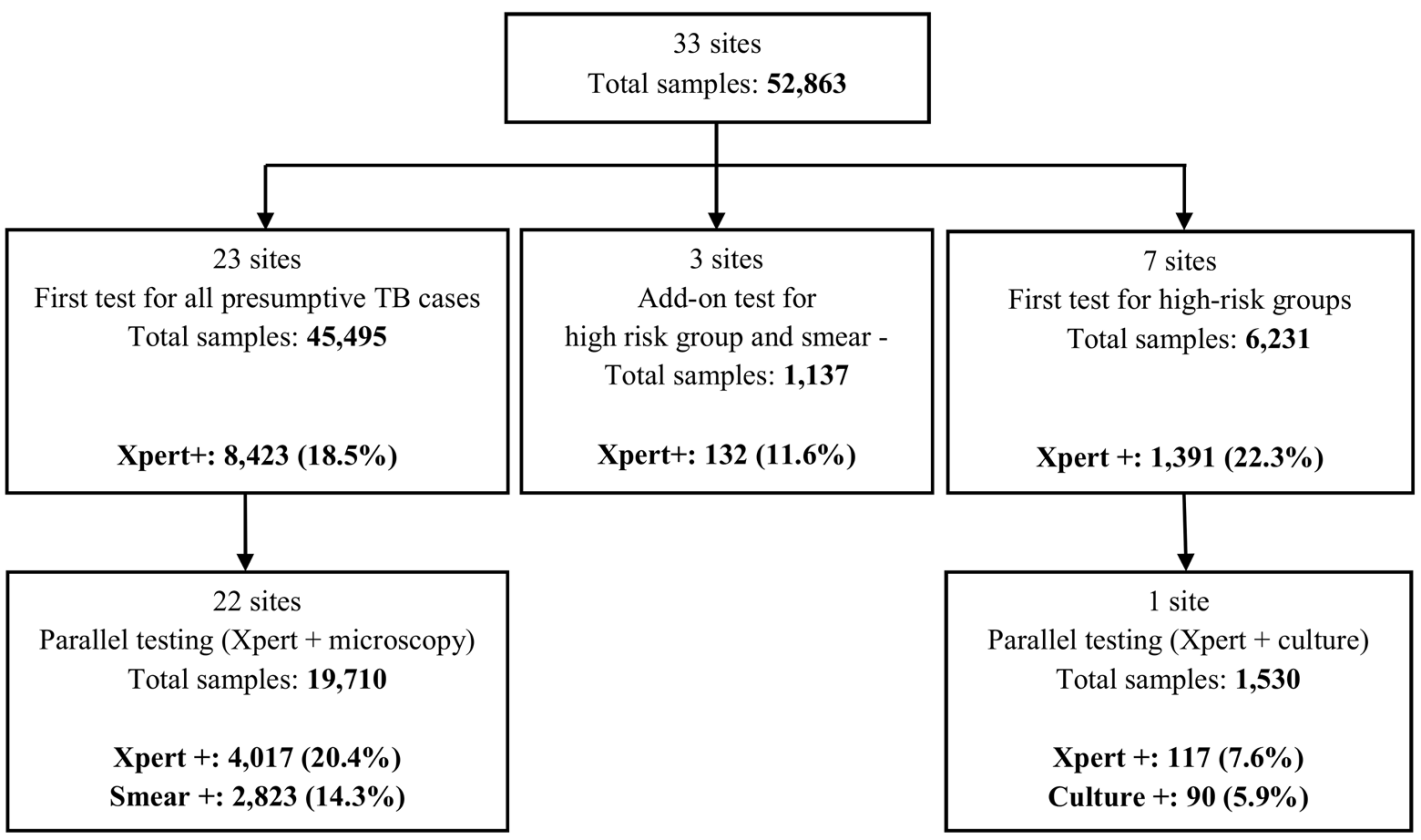
Xpert testing strategies and MTB detection.

Between April 2011 and December 2012, results for a total of 52,863 Xpert tests were reported (Fig 1). Of the total 45,495 tests performed as the initial diagnostic test, the average MTB positivity rate was 18.5% **(Table 2)**, with large variations between sites [range 9.7–43.8%].

**Table 2:** Detection of MTB by Xpert in 23 sites using Xpert as first test.

Project	Positive	Negative	Inconclusive	Total
	n (%)	n (%)	n (%)	
CAR, Zemio	9 (18.4)	28 (57.1)	12 (24.5)	49
DRC, Kinshasa	144 (17.3)	656 (78.8)	33 (4.0)	833
Kenya, Kibera	437 (28.6)	1,023 (67.0)	67 (4.4)	1,527
Kenya, Mathare	589 (22.3)	1,668 (63.2)	381 (14.4)	2,638
Kyrgyzstan, Bishkek	309 (30.9)	641 (64.1)	50 (5.0)	1
Kyrgyzstan, Kara Suu	72 (23.8)	193 (63.7)	38 (12.5)	303
Lesotho, Roma	171 (19.2)	666 (74.8)	53 (6.0)	890
Mozambique, Mavalane	782 (31.1)	1,528 (60.8)	204 (8.1)	2,514
Mozambique, Moatize	487 (21.6)	1,531 (67.8)	241 (10.7)	2,259
Russia, Grozny	315 (43.8)	399 (55.5)	5 (0.7)	719
South Africa, Eshowe	1,309 (18.6)	5,542 (78.8)	181 (2.6)	7,032
South Africa, Mbongolwane	379 (9.7)	3,412 (87.7)	101 (2.6)	3,892
Swaziland, Hlatikulu	243 (13.9)	1,375 (78.5)	133 (7.6)	1,751
Swaziland, Mankayane	330 (12.8)	2,113 (81.9)	136 (5.3)	2,579
Swaziland, Matsanjeni	103 (13.6)	617 (81.7)	35 (4.6)	755
Swaziland, Matsapha	758 (13.9)	4392 (80.7)	294 (5.4)	5,444
Swaziland, Nhlangano	441 (13.9)	2,542 (80.1)	191 (6.0)	3,174
Uzbekistan, Chimbay	64 (19.5)	251 (76.5)	13 (4.0)	328
Zimbabwe, Epworth	627 (23.1)	1,882 (69.3)	205 (7.6)	2,714
Zimbabwe, Birchenough	195 (18.5)	802 (76.2)	55 (5.2)	1,052
Zimbabwe, Gokwe	5 (26.3)	9 (47.4)	5 (26.3)	19
Zimbabwe, Gutu	67 (18.3)	298 (81.4)	1 (0.3)	366
Zimbabwe, Murambinda	587 (16.1)	2,651 (72.5)	419 (11.5)	3,657
TOTAL	8,423 (18.5)	34,219 (75.2)	2,853 (6.3)	45,495

Of the 6,231 tests performed as the initial test only for high risk groups, the average MTB positivity rate was 22.3%, again with wide variation between sites [range 13.3–66.7%] **(Table 3)**.

**Table 3:** Detection of MTB in 7 sites using Xpert as first test in high risk groups.

Project	MTB Positive	MTB Negative	MTB Inconclusive	Total
	n (%)	n (%)	n (%)	
Colombia, Buenaventura	207 (42.2)	273 (55.7)	10 (2.0)	490
Cambodia, KC	383 (15.3)	1,884(75.3)	234 (9,4)	2,501
Georgia, Abkhazia	163 (25.7)	439 (69.1)	33 (5.2)	635
India, Manipur	14 (66.7)	3 (14.3)	4 (19.0)	21
Myanmar, Yangon	419 (38.6)	646 (59.5)	21 (1.9)	1,086
Somalia, Galcayo	8 (57.1)	6 (42.9)	0 (0.0)	14
Uzbekistan, Nukus	197 (13.3)	1,206 (81.3)	81 (5.5)	1,484
TOTAL	1,391 (22.3)	4,457 (71.5)	383 (6.1)	6,231

At the three sites where Xpert was used as the first test for high risk groups or as an add-on test to microscopy, of the 1,137 tests performed, the average positivity rate was 11.6% [range 4.1–14.1%] **(Table 4).**

**Table 4:** Detection of MTB in 3 sites using Xpert as first test in high risk groups and as add-on test to microscopy for smear negative presumed TB.

Project	MTB Positive n (%)	MTB Negative n (%)	MTB Inconclusive n (%)	Total
Kenya, Homa Bay	82 (14.1)	460 (79.3)	38 (6.6)	580
Malawi, Chiradzulu	40 (12.8)	224 (71.8)	48 (15.4)	312
Malawi, Thyolo	10 (4.1)	216 (88.2)	19 (7.8)	245
TOTAL	132 (11.6)	900 (79.1)	105(9.2)	1,137

Of 14 sites with data available for comparison, both for Xpert and microscopy the MTB positivity rate decreased significantly from the early implementation phase to the routine phase, for Xpert from 22.9% (818/3576) to 19.7% (2656/13479) (p = 0.002), while the smear positivity rate decreased from 16.9% (606/3576) to 14% (1885/13479) (p = 0.0002).

Twenty-four sites reported results from 1,278 samples collected from children, representing 2.4% of the total 52,863 samples tested. Of these, 61 were tested positive (4.7%) with Xpert.

### Detection of rifampicin resistance

The rifampicin resistance detection rate among MTB-positive samples ranged from 0 to 41.4% (Table 5) across all 33 sites. Overall, 123 of 9,929 (1.2%) Xpert-positive samples generated rifampicin-indeterminate results. Microscopy results were reported for 79 of the 123 Xpert rifampicin-indeterminate results, of which 63 (79.7%) were found to be smear-negative.

**Table 5:** Detection of rifampicin resistance by Xpert according to MDR-TB prevalence.

MDR-TB prevalence	Country	MTB+/RIF resistant	MTB+/RIF susceptible	MTB+/RIF indeterminate	TOTAL MTB+
		n (%)	n (%)	n (%)	n
	Colombia	23 (11.1)	184 (88.9)	0 (0.0)	207
	Georgia	30 (18.4)	130 (79.8)	3 (1.8)	163
	India	5 (35.7)	9 (64.3)	0 (0.0)	14
	Kyrgyzstan	137 (36.0)	237 (62.2)	7 (1.8)	381
	Lesotho	13 (7.6)	158 (92.4)	0 (0.0)	171
High	Myanmar	71 (16.9)	339 (80.9)	9 (2.1)	419
	Russia	80 (25.4)	234 (74.3)	1 (0.3)	315
	South Africa	235 (13.9)	1,451 (86.0)	2 (0.1)	1,688
	Swaziland	197 (10.5)	1,636 (87.3)	42 (2.2)	1,875
	Uzbekistan	108 (41.4)	147 (56.3)	6 (2.3)	261
	**SUBTOTAL**	**899 (16.3)**	**4,525 (82.3)**	**70 (1.3)**	**5,494**
	DRC	17 (11.8)	122 (84.7)	5 (3.5)	144
	Cambodia	21 (5.5)	355 (92.7)	7 (1.8)	383
	Kenya	59 (5.3)	1,031 (93.1)	18 (1.6)	1,108
Low	Malawi	0 (0.0)	49 (98.0)	1 (2.0)	50
	Mozambique	99 (7.8)	1,163 (91.6)	7 (0.6)	1,269
	Zimbabwe	73 (4.9)	1,393 (94.1)	15 (1.0)	1,481
	**SUBTOTAL**	**269 (6.1)**	**4,113 (92.7)**	**53 (1.2)**	**4,435**
	**TOTAL**	**1,168 (11.7)**	**8,638 (86.9)**	**123 (1.2)**	**9,929**

### Xpert results compared to sputum smear microscopy

Of the 22 sites that performed Xpert and microscopy in parallel for all presumptive TB cases, fluorescence microscopy (FM) and Ziehl-Neelsen (ZN) were performed in 12 and 10 sites, respectively. Of 19,710 samples tested in parallel, MTB was detected in 4,227 (21.4%) by either of the two techniques: 2,823 (14.3%) were positive by microscopy, and 4,017 (20.4%) were positive by Xpert, with a statistically significant difference (p<0.0001) and a kappa coefficient of 0.62 (Table 6). Using Xpert as an add-on test following microscopy would have resulted in an average 49.7% relative gain in bacteriologically-confirmed TB, while replacing microscopy with Xpert as the first test in the diagnostic algorithm would have increased the laboratory detection of MTB compared to microscopy by 42.3%.

**Table 6:** Results of Xpert compared to smear microscopy.

	Xpert				
		Positive	Negative	Inconclusive	Total
**Smear microscopy**	Positive	2,613	73	137	2,823
	Negative	1,404	14,143	1,340	16,887
	TOTAL	4,017	14,216	1,477	19,710

The relative gain of Xpert as add-on test versus microscopy varied widely between sites, from 9.7% to 110.4%, and was significantly higher in sites performing ZN (77.5%) compared to FM microscopy (39.6%, p<0.001) (Table 7).

**Table 7:** Relative gain by project for Xpert used as add-on test.

		Xp+/ Sm-	Xp +/ sm+	Sm+	Relative gain
**FM**	Kenya, Mathare	58	535	596	9,7%
	Kenya, Kibera	54	239	240	22,5%
	Malawi, Chiradzulu	13	27	27	48,1%
	Zimbabwe, Epworth	210	410	428	49,1%
	DRC, Kinshasa	46	82	88	52,3%
	Kenya, Homa Bay	29	48	53	54,7%
	Zimbabwe, Murambinda	57	88	101	56,4%
	Swaziland, Hlatikulu	93	148	154	60,4%
	Swaziland, Nhlangano	177	245	269	65,8%
	Zimbabwe, Birchenough	28	38	39	71,8%
	Swaziland, Matsanjeni	47	52	56	83,9%
	Zimbabwe, Gokwe	2	2	2	100,0%
	TOTAL	814	1914	2053	39,6%
**ZN**	CAR, Zemio	2	7	11	18,2%
	Russia, Grozny	103	190	213	48,4%
	Zimbabwe, Gutu	25	42	43	58,1%
	Mozambique, Mavalane	46	64	67	68,7%
	Kyrgystan, Bishkek	46	46	63	73,0%
	Swaziland, Mankayane	14	16	19	73,7%
	Uzbekystan, Chimbay	31	29	35	88,6%
	Lesotho, Roma	20	20	20	100,0%
	Mozambique, Moatize	250	230	242	103,3%
	Swaziland, Matsapha	53	46	48	110,4%
	TOTAL	590	690	761	77,5%

### Results of Xpert compared to culture

Of the three sites performing Xpert in parallel with culture, only Cambodia provided laboratory results for a total of 1,530 smear-negative samples tested in parallel with Xpert and MGIT **(Table 8**). Of a total of 157 TB patients detected by either test, culture detected 90 (57.3%) versus 117 (74.5%) detected by Xpert.

**Table 8:** Detection of MTB by Xpert compared to MGIT culture in smear-negative samples.

	Culture				
Xpert	Positive	NTM	Negative	Contaminated	Total
MTB positive	50	8	38	21	117
MTB negative	33	99	1009	138	1,279
MTB inconclusive	7	16	87	24	134
TOTAL	90	123	1,134	183	1,530

NTM: non-tuberculous mycobacteria

### Xpert inconclusive results, module replacement and errors

Of the total 52,863 samples tested, 3,341 (6.3%) generated inconclusive results. The proportion varied between projects, with a median of 5.7% [range 0–26.3%]. Only six sites reported a level of inconclusive results below 3%.

To analyse factors that might have influenced the proportion of inconclusive results, we analysed their frequency by level of facility where Xpert was implemented, the module replacement, the cartridge version used (G4 versus G3), and staff experience in performing the test (routine versus implementation phase).

When analysed by level of facility, the proportion of inconclusive results was 2.5% (73/2965) at regional level, 6.4% (2680/42021) at district and sub-district level, 7.8% (538/6877) at peripheral level and 5.0% (50/1000) in the penitentiary system (p<0.001).

In total, 12 sites underwent module replacement due to high error rates, with an average of 8.2% (2332/27382) inconclusive results versus 4.4% (1109/25481) for projects that did not change module (p<0.001). However, none of the 12 projects reached a rate of inconclusive results below 3% after module replacement.

Of the 33 sites, 27 had a period of activity longer than 4 months, covering both implementation and routine phases, allowing a comparison of outcomes during these two time periods. Overall, the proportion of inconclusive results was significantly higher during the implementation compared with the routine activity phase (8.0% vs 5.8%, p<0.001).

The proportion of inconclusive results was 7.4% using the G3 cartridge and 5.8% using the G4 cartridge (p<0.001).

Stratification by phase and cartridge version showed that the phase had an impact only when the G3 cartridge was used, while the cartridge change from G3 to G4 reduced the proportion of inconclusive results irrespective of the phase of implementation (Table 9).

**Table 9:** Inconclusive Xpert results by implementation phase and cartridge version.

	Xpert inconclusive results			
Cartridge version	Phase 1	Phase 2	Total	
G3	8.8% (557/6,360)	6.9% (1,199/17,278)	7.4% (1756/23,638)	P<0.001
G4	4.7% (69/1,466)	5.1% (1,365/26,845)	5.1% (1,434/28,311)	P = 0.54
TOTAL	8.5% (626/7,826)	5.8% (2,564/44,123)	6.1% (3,190/51,949)	
	p<0.001	p<0.001		

Thirty projects reported information on the frequency of error 5011. For G3, error 5011 accounted for 425 of 888 errors (47.9%), and 30.8% of the 1381 inconclusive results with this cartridge, while for G4 it represented 379 of 978 errors (38.8%) and 30.1% of the 1260 inconclusive results with G4. Although the decrease was significant (p = 0.03), the frequency of error 5011 remained high.

### Identifying key lessons learned

In all sites logistical requirements were in place before implementation of the test; they were either already in place, or implemented expressly for Xpert introduction. Twenty eight questionnaires on implementation issues were received, providing information for all implementing sites (**Table 10)**. Three countries with multiple sites, Swaziland, Zimbabwe and South Africa, completed a single questionnaire with combined country information. Projects reported installation of air conditioning as one of the main logistical interventions (54%), followed by installation of a generator (39%), while the majority of the laboratories were already equipped with a biosafety cabinet prior to Xpert implementation (89%).

**Table 10:** Quantitative results from the lessons learned questionnaire (n = 28).

Infrastructure	Yes	No
	n (%)	
Laboratory renovation required	5 (18)	23 (82)
Air conditioning installed for test implementation	15 (54)	13 (46)
Generator installed for test implementation	11 (39)	17 (61)
Installation biosafety cabinet for test implementation	3 (11)	25 (89)
**Equipment performance**		
Failed installation check (one module per machine)	2 (7)	26 (93)
Experienced performance problems	9 (32) ^1^	21 68)
**Assay performance**		
Staff computer training required	10 (36)	18 (64)
High error rates reported to Cepheid	14 (50)	14 (50)
Modules replaced on advice of Cepheid	11 (39)	17 (61)
**Module calibration**		
Module exchange-based calibration procedure followed	11 (39) ^2^	17 (61)
**Impact on programmes**		
Sputum collection strategy changed	7 (25)	21 (75)
**Overall impressions**		
Satisfaction with the system due to: simplicity of procedure	17 (61)	11 (39)
Speed of assay	6 (21)	22 (79)
Increased sensitivity cf. smear microscopy	5 (18)	23 (82)
Frustrations due to: high error rates	17 (61)	11 (39)
Lack of Russian-language software	3 (11)	25 (89)
Lack of isoniazid resistance detection	2 (7)	26 (93)
**Most positive aspects**		
On-site rifampicin resistance detection	11 (39)	17 (61)
Increased sensitivity for tuberculosis detection	12 (43)	16 (57)
Speed to results	2 (7)	26 (93)
Simplicity of use	3 (11)	25 (89)

1. 5/9 experienced barcode scanning problems; 2/9 sites had GeneXpert machine failure when the ambient temperature exceeded 30°C; 1/9 had a cartridge stuck in a module.

2. This process went smoothly for 8/11; 2/11 experienced customs problems, and 1/11 experienced a long delay in shipment of replacement modules.

High rates of inconclusive results were reported as one of the main limitations by almost half of the respondents. Fourteen respondents mentioned having contacted the manufacturer (Cepheid) specifically regarding the high rate of inconclusive results. Eleven respondents reported having modules replaced, upon the manufacturer’s advice, for a total of 21 replaced modules. Two modules also failed the installation check. In total, 15% [23/152] of all modules initially distributed were replaced.

Seven of 28 respondents reported changing their sputum collection strategy as a result of implementing Xpert, with two sites moving from the collection of three to two samples, and three sites from two to one sample. Two sites adopted spot-spot collection cf. spot-morning.

Lack of the user manual and software in Russian at the time of implementation (later addressed by the manufacturer) hindered Xpert usage in some settings.

Limited internet access was a barrier for annual calibration. Other challenges included the need for basic computer training in one third of the sites, where microscopists were not sufficiently familiar with their use.

All respondents reported being generally satisfied with the system. However, some commented that discordant results between Xpert and culture made interpretation of results difficult, that bloody sputum resulted in inconclusive results, and that viruses occasionally infected the computer used with the system.

## Discussion

As countries embark on the implementation and scale up of the new Xpert technology, there is an increasing need to document and share the programmatic and operational lessons emerging from this logistically-intensive activity, especially across different settings in resource-constrained contexts.

Our experience showed that Xpert significantly contributed to TB detection when used as the first diagnostic test for all presumptive TB in parallel with microscopy, and that it would have led to a high relative gain if used as a replacement test. At one site Xpert and culture on MGIT equally contributed to detection of TB among smear-negative presumptive TB patients, and overall the test contributed to the detection of TB in paediatric samples, although analysis on this point is limited by the relatively small paediatric sample size. High rates of inconclusive results represented one of the major challenges, related to various factors including technical issues and staff experience. Xpert implementation required consistent laboratory support with costly logistical interventions.

In our study, when compared to microscopy, the Xpert relative gain varied according to the technique used, (i.e. higher compared with ZN cf. FM). In general Xpert relative gain varied between projects. Apart from the program in Central African Republic, for which the number of tests performed was very low, two sites in Kenya, Kibera and Mathare, showed lower relative gain. These results may be explained by the different testing strategies implemented and the heterogeneous epidemiological contexts in which the test was deployed. For example, if TB patients tend to wait before seeking medical care, they may have high bacillary loads which can be detected by both smear microscopy and Xpert. This might explain the comparatively low relative gain in the Mathare and Kibera projects, which are located in a slum, where the smear positivity rate was higher than for all other sites included in the study, which suggests that patients generally sought care at an advanced stage of the disease.

The positivity rate decreased significantly from the implementation to the routine phase. The positivity rate in the routine phase was similar to that reported by South Africa (16%), India (20%) and the TB REACH multicentric study (15%). [17,18,11] The decrease over time could be explained by an increased number of presumptive TB patients tested with Xpert over time, as also observed by other authors. [19]

Our results show that replacing microscopy with Xpert would have resulted in fewer cases detected rather than performing Xpert as an add-on test following microscopy, which may be explained by several factors. Firstly, among cases detected by microscopy but not Xpert, the majority were due to Xpert inconclusive results. In these cases repeating the test on a new sample may have produced a positive Xpert result; however, these results were not available, thus the proportion of cases detected by Xpert may be underestimated.[20] Secondly, Xpert negative, smear positive results could be due to the presence of non-tuberculosis mycobacteria (NTM) that can be detected by microscopy but are reported as negative by Xpert, as the assay is highly specific for the detection of *M*. *tuberculosis* complex, and cross-reaction with NTM has not been reported. Thirdly, the quality of microscopy in these sites was high, performed under regularly supervised and controlled conditions. On the other hand, microscopy performance could also be underestimated, since the result of only one specimen was considered, whereas microscopy investigation is normally based on testing at least two samples. [21] It is known that microscopic investigation of a second sample can increase case detection by 10–14%. [22] Considering the increased detection from testing a second sample, the incremental yield of Xpert compared to conventional microscopy would have still accounted for an estimated 18.6%–24.6%. While case detection would increase by the use of Xpert as an add-on test to smear negative samples, the low positivity rate of microscopy shows that 86% of the cases not detected by microscopy would still require testing with Xpert. This approach would represent a very high workload and investment compared to directly performing Xpert as the first diagnostic test, and should be carefully considered.

A multicentric field demonstration study carried out in six countries reported that a single Xpert test detected 90.3% of TB cases which were bacteriologically confirmed by liquid culture. [8] In our study, the comparison between Xpert and culture was only possible at one site, where the sample was tested in parallel with Xpert and the MGIT automated system. At this site, Xpert detected only 55.6% of cases confirmed by culture, which is lower than reported elsewhere [8], but comparable to values reported by Theron [23], who also reported a per sample analysis; while Boehme compared Xpert positivity to final culture results obtained by multiple testing, which may decrease Xpert positive/culture negative results. However, Xpert also detected a substantial number of cases that were missed by culture, either because of contamination, or harsh decontamination leading to negative results, or possible mixed infection with NTM, which would have been misclassified by culture as NTM positive.

For rifampicin resistance detection, rates of indeterminate results were lower than the 2.4% reported in a field demonstration study [8] and comparable with results reported by the TB REACH study.[11] As expected, indeterminate results were found mostly in smear negative samples, with low bacillary load; however, almost 20% occurred in smear-positive samples.

Overall the number of children screened with Xpert for TB was low at all sites, possibly due to the difficulty of obtaining an adequate sample for laboratory confirmation. Among the children tested, the detection of TB using Xpert was lower than in other studies.[24] However, in our study only one sample was tested per patient, while other authors report that to increase detection in children, algorithms including collection of samples from different body sites (e.g. gastric aspirates) should be included. [25]

The rate of inconclusive results in the first multi-country feasibility study conducted in seven countries was below 3% [8]; South Africa achieved a similar rate.[17] However, in MSF-supported sites, half of the 33 sites had rates of more than double this benchmark. The TB REACH multicentric study reported rates in agreement with ours (10.6%) [11], possibly due to the related routine implementation conditions, compared with the Boehme evaluation study. This value is also comparable or higher than rates for interpretable results with other validated tests, such as Genotype MTBDRplus assay (92%) and conventional methods (78%). [26]

The high rate of inconclusive results in our study was a source of frustration, due to having to collect replacement samples from some patients, results being delayed, and the expense of having to use multiple cartridges for one patient. This unforeseen rate of usage in some sites led to stock rupture, therefore having a detrimental impact on patient diagnosis.

In MSF projects the algorithm included retesting Xpert for patients with inconclusive results, when possible using the leftover sample, or a newly collected sample, but results from retesting were not available and are not included in this analysis. Repeating Xpert testing for inconclusive results is reported to resolve inconclusive results [5], so this procedure should be included in the routine diagnostic algorithm, and results of retesting should be collected when possible.

Inconclusive results can occur for different reasons, including incorrect manipulation of the samples. [27] Our results suggested that several aspects may have been correlated with inconclusive results, showing that aside from technical limitations, which were partially resolved by module replacement and cartridge version, staff experience in performing the test contributed to decreasing the rate of inconclusive results. In a report published by FIND it is stated that due to the launch of the G4 cartridge, error 5011 was virtually eliminated. [15] In our experience, implementation of the new cartridge across our sites decreased the occurrence of error 5011; however it was still reported by the laboratories on occasion. Due to the lack of information regarding the distinction between the type of inconclusive result and error codes other than 5011, this retrospective analysis of routinely collected data could not account for other factors influencing the rate of inconclusive results, such as turnover of the staff, temperature fluctuations in the laboratory or power supply interruptions.

The Xpert system was initially described as easy to perform, requiring minimal training and set up, including in peripheral settings. However, in our experience the device was not uniformly easy to install and operate. Its implementation required costly interventions, including provision of air conditioning, provision of uninterrupted electricity and internet connection for calibration. Until a more robust system is available, these extra costs need to be taken into account prior to the decision to introduce the test. The feedback from users was overall positive, mainly due to the simplicity of the procedure. However, aside from logistical interventions, implementation required regular technical support, including training in results interpretation, which had to be adapted to the level of the facility, such as in the case of reference laboratories due to discordant results with culture techniques. Language issues, which initially hindered implementation in some sites, were eventually addressed by the manufacturer.

This study presents some limitations. In this per sample analysis the comparison of Xpert versus microscopy was based on investigation of one sample per patient, while routinely microscopy is performed on multiple samples and Xpert is repeated for inconclusive cases. The data analysis did not include results from repeated Xpert testing for inconclusive results, which may have increased the positivity rate for this test. The use of aggregated data collected routinely prevented us from undertaking a more precise and accurate investigation of the factors associated with proportion of inconclusive results.

## Conclusion

The implementation of Xpert in diverse clinical settings was feasible and led to a significant increase in bacteriologically-confirmed pulmonary TB both for Xpert as first test, and as an add-on test. The choice of the best strategy should take into account the epidemiological setting, including prevalence of NTM, and the test cost which may represent a limitation in resource-constrained settings. However, the estimation of the cost should take into account that the major investment is often represented by the logistical support required for installation of the system, while the higher test cost compared to microscopy is offset by the higher sensitivity and specificity compared to microscopy'.

In our experience the system was far from a “plug and play” device. High numbers of inconclusive results represented an extra expense. Significant infrastructure requirements, training, technical support and experience were indispensable to decrease errors and achieve good routine results, and time was needed for programmes to become more effective in applying sample collection strategies. To further decentralize diagnosis of drug-sensitive and drug-resistant TB strains, more robust, simpler technologies which are well-adapted to low-resource settings are still needed. In addition, as the GeneXpert system is now relatively widespread in many high-burden countries, development of a cartridge incorporating resistance detection for other drugs could boost the fight against drug-resistant tuberculosis.
